# Total Arch Replacement With Frozen Elephant Trunk Using a NEW “Brain-Heart-First” Strategy for Acute DeBakey Type I Aortic Dissection Can Be Performed Under Mild Hypothermia (≥30°C) With Satisfactory Outcomes

**DOI:** 10.3389/fcvm.2022.806822

**Published:** 2022-02-08

**Authors:** Kangjun Shen, Ling Tan, Hao Tang, Xinmin Zhou, Jun Xiao, Dongshu Xie, Jingyu Li, Yichuan Chen

**Affiliations:** ^1^Department of Cardiovascular Surgery, The Second Xiangya Hospital of Central South University, Changsha, China; ^2^Department of Cardiac Surgery, Union Hospital, Fujian Medical University, Fuzhou, China

**Keywords:** total arch replacement, frozen elephant trunk, mild hypothermia, heart-brain-first, acute DeBakey type I aortic dissection

## Abstract

**Background:**

Total arch replacement (TAR) with Frozen elephant trunk (FET) treatment of acute DeBakey type I aortic dissection (ADIAD) is complicated, carries a high complication/mortality risk and remains controversial on the optimal hypothermic level, cerebral perfusion and visceral organ protection strategy. We developed a new strategy named “Brain-Heart-first” in which the surgical procedures and the management of cardiac perfusion/cerebral protection during Cardiopulmonary bypass (CPB) were redesigned, and TAR with FET technique can be performed under mild hypothermia with satisfactory outcomes.

**Objective:**

Our aims were to describe a new surgical strategy under mild hypothermia (≥30°C) for the treatment of ADIAD and to report the operative outcomes of 215 patients.

**Methods:**

We conducted a retrospective analysis of 215 consecutive cases of ADIAD treated with our new strategy.

**Results:**

The durations of CPB, aortic cross-clamping, antegrade cerebral perfusion, operation, mechanical ventilation support, and Intensive Care Unit stay were 139.7 ± 52.3 min, 55.6 ± 27.4 min, 14.1 ± 3.1 min, 6.0 ± 1.7 h, 40.0 h and 4.0 d, respectively. The 30-day mortality was 9.8%, with cerebral stroke occurring in nine patients (4.2%), paraplegia in one patient (0.5%) and postoperative renal injury requiring dialysis in 21 patients (9.8%). The blood transfusion of red blood cells and fresh frozen plasma during surgery and the first 24 h after surgery was 4.0 u and 200.0 ml, respectively.

**Conclusions:**

The Brain-Heart-first strategy can be widely used with low technical and resource requirements and provides a safe alternative for conventional TAR with FET technique in ADIAD patients with satisfactory operative results.

## Introduction

Although the application of total arch replacement (TAR) combined with frozen elephant trunk (FET) technique in acute DeBakey type I aortic dissection (ADIAD) still remains controversial, it has evolved as an accepted choice for patients with aortic disease involving the aortic arch and supra-arch branches (including ADIAD) (1–5). However, this technique is an extended, complex, time-consuming and skill-demanding surgical strategy (1–5). Deep hypothermic circulatory arrest (DHCA) seems to be necessary, and therefore different strategies of cerebral perfusion (unilateral/bilateral and antegrade/retrograde cerebral perfusion) are developed (2–6). There is no consensus on the optimal hypothermia level (mild, moderate, or deep), cerebral perfusion, and visceral organ protection currently. Various modifications of the technique [hybrid procedures or new devices, such as triple-branched stent graft (7) and custom-made E-Vita graft (8)] have been reported to dispense the constraint of DHCA, decrease the operative invasiveness, and increase safety by optimizing or simplifying the procedures (6–13). However, the application of these modified techniques is limited due to their inherent deficiencies, e.g., restricted surgical indications, requirement for well-equipped hybrid surgical units, and necessity of validation through long-term clinical trials (6–13). Conventional TAR with FET technique is still the mainstream choice for ADIAD. Based on our preliminary improvement on the conventional technique (the Arch-first strategy) (14), we further optimized and redesigned the conventional TAR with FET procedure to decrease the difficulty in operation, shorten operation time, reduce postoperative complications and 30-day mortality. This optimized procedure can be performed under mild hypothermia (≥30°C) and was designated as the “Brain-Heart-first” strategy. In the present study, we described the modifications and surgical outcomes retrospectively.

## Materials and Methods

### Patient Enrollment

A total of 215 consecutive patients with ADIAD who underwent treatment using our new Brain-Heart-first strategy under mild hypothermia (≥30°C) by a single surgical team were enrolled between May 1, 2017 and December 31, 2020. The preoperative characteristics of patients are listed in Table 1.

**Table 1:** Characteristics of 215 consecutive patients with ADIAD who underwent treatment using the Brain-Heart-first strategy.

**Variable**	**The whole cohort**
Male gender [*n* (%)]	168 (78.1)
Age (years)	50.5 ± 11.5
Hypertension [*n* (%)]	153 (71.2)
Diabetes [*n* (%)]	4 (1.9)
Marfan syndrome [*n* (%)]	7 (3.3)
Bicuspid aortic valve [*n* (%)]	2 (0.9)
Coronary heart disease [*n* (%)]	30 (14.0)
Cardiac valve surgery [*n* (%)]	3 (1.4)
Thoracic endovascular repair [*n* (%)]	5 (2.3)
Percutaneous coronary intervention [*n* (%)]	5 (2.3)
Preoperative renal injury [*n* (%)]	39 (18.1)

*Preoperative renal injury was defined with a creatinine clearance rate < 50 ml/min*.

### Surgical Procedures

**Figure 1:**
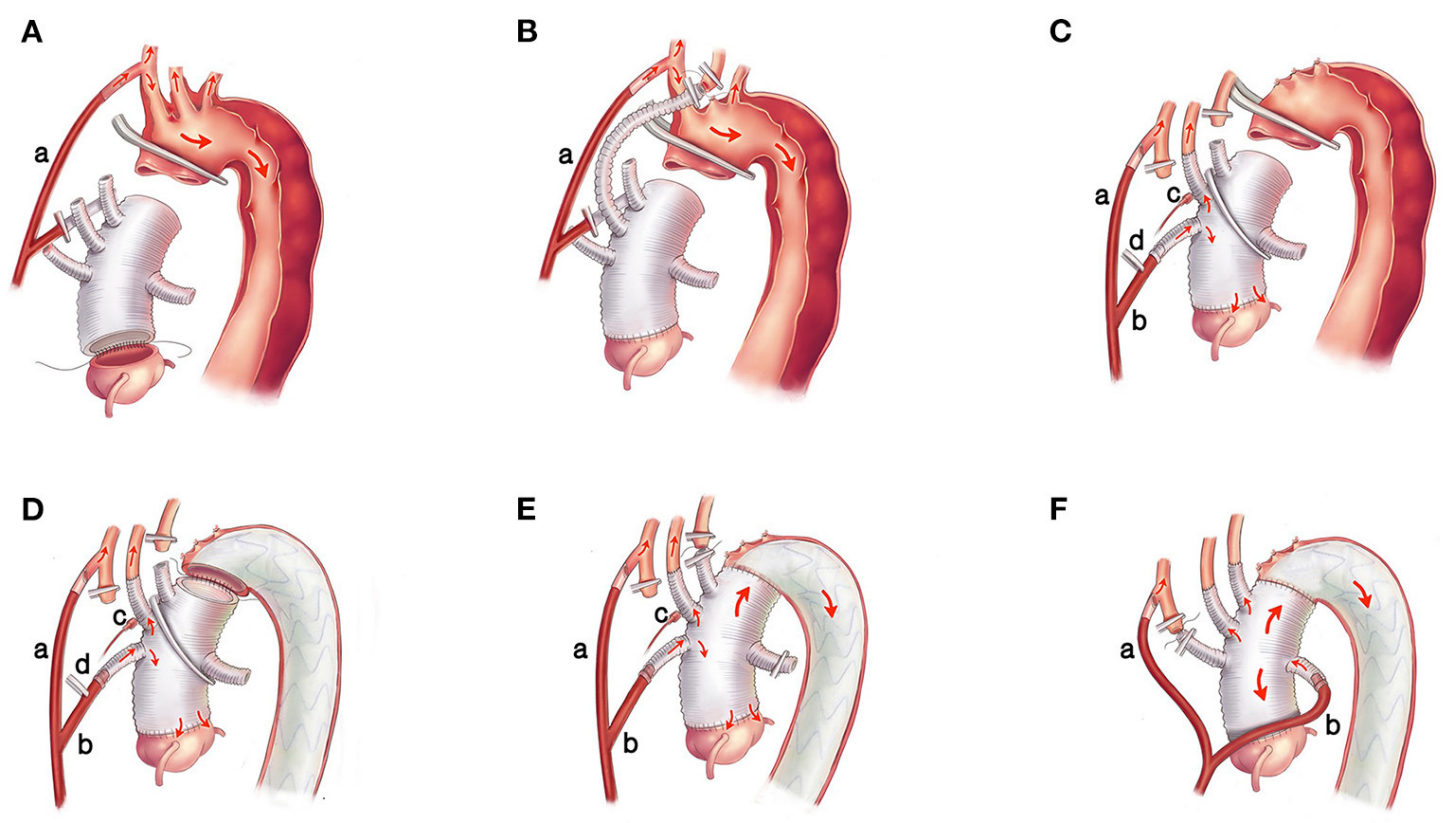
The surgical procedures of the Brain-Heart-first strategy. **(A)** One end of the Y-shaped arterial perfusion cannula (a) is inserted into the right axillary artery to supply the body. The ascending aorta is replaced using a 4-branched graft, and the aortic root/valve/coronary is reconstructed or replaced if necessary. **(B)** The LCCA is anastomosed with the 2nd branch of the 4-branched graft, and the IA and LSA supply the cerebral circulation system temporarily. **(C)** The other end of the Y-shaped arterial perfusion cannula (b) is connected with the 1st branch of the 4-branched graft, and the trunk of the graft is cross-clamped between the 2nd and the 3rd branches after de-airing adequately. Continuous perfusion to the heart and LCCA is achieved. The blood pressure of the LCCA is monitored by an arterial pressure catheter (c) and adjusted by partially clamping the perfusion tube in the 1st branch (d). **(D)** The stented graft is inserted into the true lumen of the descending aorta and anastomosed with the 4-branched graft. **(E)** The perfusion in the lower body is resumed, and the LSA is anastomosed with the 3rd branch of the 4-branched graft. **(F)** The perfusion tube is transferred from the first branch to the 4th branch of the 4-branched graft, and the IA is anastomosed with the 1st branch. LCCA, left common carotid artery; IA, innominate artery; LSA, left subclavian artery.

General anesthesia was administered. Two atrial pressure lines (1 left radial and 1 left/right pedal) and a transesophageal echocardiographic monitoring probe were placed routinely. Bilateral regional cerebral oxygen saturation was monitored using near-infrared spectroscopy (NIRS) (CAS Medical systems, Inc., Branford, CT, USA). Cardiopulmonary bypass (CPB) was instituted through the right atria to the right axillary artery by cannulation (one end of the Y-shaped arterial perfusion cannula is inserted into the right axillary artery). Femoral artery cannulation was performed if necessary, e.g., when the pumping pressure was >250 mmHg. CPB was initiated with a flow rate of 2.0-2.4 L/m^2^/min, and systemic cooling was commenced to induce mild hypothermia. During the cooling phase, the ascending aorta was replaced using a 4-branched graft, and the aorta root/valve/coronary was reconstructed or replaced if necessary. Approximately 4-5cm length was reserved at the proximal end of the 4-branched graft to cross-clamping during the potential reoperation (Figure 1A).

After reconstruction of the ascending aorta and/or aortic root, the left common carotid artery (LCCA) was anastomosed with the second branch of the 4-branched graft, and the innominate artery (IA) and the left subclavian artery (LSA) supplied the cerebral circulation system temporarily (Figure 1B). Then, the other end of the Y-shaped arterial perfusion cannula was connected with the first branch of the 4-branched graft, and the trunk of the 4-branched graft was cross-clamped between the second and the third branches to obtain enough operation space to complete the distal anastomosis between the trunk of 4-branched graft and the stented graft. The LCCA was then de-clamped after de-airing, and continuous perfusion to the heart and the LCCA was achieved (Figure 1C). The blood pressure of the LCCA was monitored by an arterial pressure catheter in the second branch. Then the pressure of LCCA was adjusted to be equal to the blood pressure of the left radial artery by partially clamping the perfusion tube in the first branch to balance the cerebral flow rate and avoid excessive irrigation (Figure 1C). After that, the LSA was cross-clamped, ligatured, and cut off. The perfusion flow rate was decreased to 1.0-1.2 L/m^2^/min and the IA was cross-clamped, ligatured, and cut off. Meanwhile, the perfusion flow rate was regulated based on pump pressure and NIRS monitoring to ensure the fluctuation of rSO_2_ and the blood pressure in the LCCA of <10% above or below the baseline. Then, the circulatory arrest of the lower body was instituted. The aorta arch was transected at the end part of zone-0 or the initial part of zone-1 (15) (Figure 1C). After clarifying the true and false lumen carefully, a 12-cm CRONUS stented graft (Microport Scientific Corp, Shanghai, China) was inserted into the true lumen of the descending aorta under direct vision, and the stented graft was anastomosed to the adventitia end-to-end with the 4-branched graft (Figure 1D). After anastomosis and de-airing, the clamps on the trunk of 4-branched graft and the clamp on the perfusion tube in the first branch were removed, and the perfusion in the lower body was resumed, the CPB flow rate was returned to 2.0-2.4 L/m^2^/min, then the LSA was anastomosed with the third branch of the 4-branche graft (Figure 1E). Rewarming was initiated when the SvO_2_ (oxygen saturation of mixed venous blood) reached >70%. After the LSA was anastomosed, the blood pressure of the left radial artery was recovered, and then the arterial pressure catheter in the second branch could be removed. The perfusion tube was transferred from the first branch to the fourth branch of the 4-branched graft (reducing the perfusion flow rate to 8 ml/kg.min within 10 s), and the IA was anastomosed with the first branch finally (Figure 1F). After rewarming, the patient was weaned from CPB, and the remainder of the procedures, including securing hemostasis and sternal closure, was routinely performed.

### Follow-Up

All survivors were contacted by telephone or direct interviews. They were followed up by means of general examination, computed tomography angiography and/or echocardiography before discharge, at 3 months after surgery, and annually thereafter.

### Data Collection

The data were collected from hospital records at the Department of Cardiovascular Surgery of The Second Xiangya Hospital of Central South University (Changsha, Hunan, China). The informed consent was signed by all patients to allow the use of their data for research, and this study was approved by the Ethics Committee of the Second Xiangya Hospital (Ethics Committee approval number: K066).

### Statistical Analysis

Data were statistically analyzed using SPSS 22.0 software (IBM Corp., Armonk, NY, USA). Continuous, normally distributed variables are presented as mean ± standard deviation. Continuous, non-normally distributed variables were shown as the median (quartile; Q_25_, Q_75_). Categorical variables are presented as absolute numbers and percentages.

## Results

### Operative Characteristics

There were no intraoperative deaths, and no aortic root wrapping or right atrium shunting was required. The operative data and concomitant procedures are summarized in Table 2. The lowest nasopharyngeal and rectal temperatures during surgery were 30.3 ± 0.9°C and 31.4 ± 0.9°C, respectively. The durations of CPB, aortic cross-clamping (ACC), LCCA cross-clamping, antegrade cerebral perfusion (ACP)/lower body arrest (LBA), and operation were 139.7 ± 52.3 min, 55.6 ± 27.4 min, 10.3 ± 2.9 min, 14.1 ± 3.1 min, and 6.0 ± 1.7 h, respectively.

**Table 2:** Operative characteristics of 215 consecutive patients with ADIAD who underwent treatment using the Brain-Heart-first strategy.

**Variable**	**The whole cohort**
**Concomitant procedure**
Aortic sinus/valve plasty [*n* (%)]	70 (32.6)
Uni-Yacoub procedure [*n* (%)]	25 (11.6)
Bentall/David procedure [*n* (%)]	22 (10.2)
Wheat procedure [*n* (%)]	5 (2.3)
CABG [*n* (%)]	20 (9.3)
**CPB characteristic**
Nasopharyngeal temperature (°C)	30.3 ± 0.9
Rectal temperature (°C)	31.4 ± 0.9
CPB time (min)	139.7 ± 52.3
Cross-clamping time (min)	55.6 ± 27.4
LCCA cross-clamping time (min)	10.3 ± 2.9
ACP/LBA time (min)	14.1 ± 3.1
Flow rate during LBA (L/m^2^/min)	1.1 ± 0.2
Pressure of LCCA (mmHg)	70.2 ± 8.0
Operation time (h)	6.0 ± 1.7

*CABG, coronary artery bypass grafting; CPB, cardiopulmonary bypass; LCCA, left common carotid artery; ACP, antegrade cerebral perfusion; LBA, lower body arrest*.

### Postoperative Outcomes

The median duration of mechanical ventilation support (MVS), Intensive Care Unit (ICU) stay, and hospitalization were 40.0 h, 4.0 days, and 16.0 days, respectively. The median amounts of red blood cells (RBC) and fresh frozen plasma (FFP) transfusion during surgery and the first 24 h after surgery were 4.0 u and 200.0 ml, respectively. The drainage within the first 24 h after surgery was 640.0 ml. Of the 215 patients, 88 (40.9%) had postoperative acute kidney injury (AKI, using Kidney Disease Improving Global Outcomes classification, KDIGO): 41 (19.1%) in stage 1, 15 (7.0%) in stage 2, and 32 (14.9%) in stage 3. Renal injury requiring dialysis occurred in 21 patients (9.8%). For the 39 patients with preoperative renal injury (creatinine clearance rate < 50 ml/min), 20 (51.3%) had their renal function recovered to normal, 3 (7.7%), 3 (7.7%), and 13 (33.3%) had stage 1, 2, 3 postoperative AKI, respectively. Postoperative cerebral stroke occurred in nine patients (4.2%), and paraplegia in one patient (0.5%). The 30-day mortality was 9.8% (21/215 patients) (Tables 3, 4).

**Table 3:** Operative outcomes of 215 consecutive patients with ADIAD who underwent treatment using the Brain-Heart-first strategy.

**Variable**	**The whole cohort**
Awaken time (h)	8.0 (4.0-18.0)
MVS (h)	40.0 (20.0-68.0)
ICU stay (days)	4.0 (2.0-7.0)
Duration of hospitalization (days)	16.0 (13.0-21.0)
RBC transfusion (u)^a^	4.0 (1.5-8.0)
FFP transfusion (ml)^a^	200.0 (0.0-550.0)
Drainage in 24 h (ml)	640.0 (470.0-940.0)
Cardiac enzyme CK-MB (NUML)	3.5 (1.7-6.2)
Cardiac enzyme TnI (NUML)	9.1 (3.5-19.5)
Postoperative acute kidney injury [*n* (%)]	88 (40.9)
Renal injury requiring dialysis [*n* (%)]	21 (9.8)
Stroke [*n* (%)]	9 (4.2)
Paraplegia [*n* (%)]	1 (0.5)
Postoperative 30-day death [*n* (%)]	21 (9.8)
Follow-up time (months)	19.9 ± 13.2
Follow-up rate (%)	99% (192/194)
Late death [*n* (%)]	16 (8.3)
Reoperation [*n* (%)]	1 (0.5)

*MVS, mechanical ventilation support; ICU, intensive care unit; RBC, red blood cells; FFP, fresh frozen plasma*.

a *During surgery and the first 24 h after surgery. NUML, multiples the upper normal limit*.

*Postoperative Renal injury was defined using the Kidney Disease Improving Global Outcomes criteria (divide into three groups)*.

**Table 4:** Patient characteristics categorized by KDIGO criteria.

**Patient population (*n*)**	**No postoperative AKI [*n* (%)]**	**Postoperative AKI [*****n*** **(%)]**		
		**Stage 1**	**Stage 2**	**Stage 3**
Whole cohort (215)	127 (59.1)	41 (19.1)	15 (7.0)	32 (14.9)
Patients with preoperative renal injury (39)	20 (51.3)	3 (7.7)	3 (7.7)	13 (33.3)

*KDIGO, Kidney Disease Improving Global Outcomes; AKI, Acute Kidney Injury*.

*Preoperative renal injury was defined creatinine clearance rate < 50 ml/min*.

### Follow-Up Data

Of the 215 patients, 21 were died in 30 days after surgery. For the other 194 patients, 2 were lost to follow-up; 192 (99.0%) completing an average follow-up of 19.9 ± 13.2 months, 16 of which died during follow-up (2 unidentified sudden deaths and 14 non- ADIAD-associated deaths), resulting in a survival rate of 91.7% (176/192 patients). There was 1 (0.5%) requirement for reoperation (aortic valve replacement due to recurrent moderate-severe aortic regurgitation during the David procedure). No other patients need reintervention for the progressive descending aorta enlargement or anastomotic leak. The patients with postoperative paraplegia who survived had their physical strength recovered to normal gradually after cerebrospinal fluid drainage.

## Discussion

The conventional TAR with FET procedure for patients with ADIAD requires deep or moderate hypothermic circulatory arrest (HCA) in the absence of hybrid procedures or new devices, which means longer cooling and rewarming phase and brings specific challenges in cardiac, neurologic, and distal organ protection (2–6). Our new surgical strategy reported satisfactory outcomes which were comparable to the published results of extensive surgical repair by the high-volume centers (2–5). Two main modifications were made in the new strategy to achieve the purpose of better protection to the brain, heart and other vital organs: one on the order of anastomosis; the other on the management of CPB (including perfusion flow, temperature and the basis of the management of CPB).

As we known, type I aortic dissection extends from the aortic root (even from the aortic valve, coronary arteries, or sinus) through the ascending aorta and aortic arch to the descending aorta. Conventionally, it was considered that the heart was far easier to be protected than the brain. Therefore, during the past decades, most surgeons give priority attention to the reconstruction of the arch and distal aorta. The reconstruction of the proximal aorta is started after the completion of the arch and distal aorta repair (3, 4), which would prolong cardiac cross-clamping time unnecessarily. In our new strategy, the order of anastomosis was modified. The proximal ascending aorta and/or aortic root would be repaired and reconstructed first, and then the LCCA was anastomosed. The major technical difficulties of this surgical procedure were the repair of the proximal aorta or aortic root actually, such as valve sparing, sinus plasty and CABG, especially in complex cases. By creative usage of the first branch as a perfusion tube, the perfusion of cardiac and LCCA was resumed after the anastomosis of the LCCA and was maintained during the reconstruction of the aortic arch and implantation of the stent graft. The procedures mentioned above would significantly shorten the duration of cardiac and cerebral ischemia (3–5). As we know, continuous cardiac perfusion could reduce myocardial damage, improve cardiac outcomes, and decrease 30-day mortality (1, 16). In the present study, the ACC time was significantly shorter, similar results were reported by other high-volume centers using hybrid procedure and new devices (7, 11, 13).

In addition to deep or moderate HCA which is essential for conventional TAR, antegrade and retrograde as well as unilateral and bilateral cerebral perfusion have been adopted with satisfactory results (17–20). However, no standards for the application of cerebral perfusion are available. Bilateral carotid arteries are known to be crucial for cerebral blood supply (17–20). In the present study, the cerebral ischemic time was minimized in patients who underwent TAR with FET using the Brain-Heart-first strategy. As mentioned above, continuous perfusion to the LCCA and the heart was achieved simultaneously through the first branch of the 4-branched graft after the LCCA was anastomosed, while the right common carotid artery had continuous perfusion *via* the right axillary artery. Therefore, sufficient cerebral perfusion through bilateral carotid arteries was achieved during the process of the arch and descending aorta repair. The only potential cerebral ischemic injury was existed in the anastomotic phase of LCCA. However, the right common carotid artery and LSA had continuous perfusion in this phase. Meanwhile, the temperature was decreased around to 30.0°C and the time for LCCA anastomosis was 10.3 ± 2.9 min, which would be safe for the cerebral perfusion. Furthermore, the blood pressure of the LCCA was monitored and was adjusted to balance the cerebral perfusion flow and avoid hypo/hyper-perfusion in bilateral common carotid arteries. As we known, a high perfusion pressure allows collateral flow from the brain to the spinal cord, with similar outcomes to those observed under a low perfusion pressure (21). In our new strategy, a high perfusion pressure (60-80 mmHg) was adopted. Different from the artificial setting in the conventional ACP (8-15 ml·kg^−1^·min^−1^) (3, 4, 7, 9, 18–20, 22), the upper body perfusion flow in the new strategy (1.00-1.23 L/m^2^/min) was produced naturally by the blood pressure, conforming with the physiological characteristics of cerebral perfusion. The perfusion flow rate during ACP/LBA was significantly increased, and effective cerebral protection was achieved as demonstrated with NIRS monitoring, without increasing the rate of stroke.

The lower body arrest time depends on the duration of anastomosis of the 4-branched graft with the FET graft. The most sensitive distal organ to ischemia is the spinal cord. Evidence showed that warmer cerebral perfusion might help to improve the collateral flow from the brain to the spinal cord, and this blood backflow may contribute to the protection of the spinal cord, which could tolerate ischemia under hypothermia at 30-32°C for 30 min (23). In our new strategy, the trunk of the 4-branched graft was cross-clamped between the second and the third branches, and the distal anastomotic area was forward to the end part of zone-0 or the initial part of zone-1, which would significantly reduce the technical difficulty of anastomosis and provide enough operation space to complete the anastomosis while ensuring sufficient heart and brain perfusion. The mean duration of anastomosis in the present study was only 14.1 ± 3.1 min without increasing the risk of postoperative paraplegia under mild hypothermia. Therefore, it is not difficult for an experienced surgeon to complete the anastomosis under this condition.

With these modifications, deep or moderate HCA is no longer needed, and the CPB time would be shortened naturally (23). It is known that a long CPB time was associated with increased postoperative complications and 30-day mortality (24, 25). In our cohort, the setting of temperature raises up to mild hypothermia. Shortened CPB and operation time were achieved, which brought satisfactory organ protection and less postoperative complications: the rates of postoperative AKI, renal injury requiring dialysis, stroke, and paraplegia was 40.9, 9.8, 4.2, and 0.5%, respectively. The rate of postoperative AKI in the present study was relatively lower than that in another large cohort in China (40.9 vs. 77.6%) (26). As we known, stage 3 AKI was associated with higher rates of major adverse events and in-hospital and 90-day deaths (26). Stage 3 postoperative AKI occurred in 14.9% of patients in the present study. For the 39 patients with preoperative renal injury, 20 (51.3%) had their renal function recovered to normal, and only 13 (33.3%) had stage 3 postoperative AKI, demonstrating the safety and effectiveness of the Brain-Heart-first strategy. The clotting disorders related to prolonged CPB was also alleviated. The amounts of RBC and FFP transfusion during surgery and within 24 h after surgery were 4.0 u and 200.0 ml, respectively; the drainage within 24 h after surgery was 640 ml. Furthermore, during follow-up, the reintervention rate was relatively low. Only 1 patient (0.5%) who underwent the David procedure in the aortic root needed reintervention for moderate-severe aortic valve insufficiency. No other patients needed reintervention for the progressive distal aorta enlargement or anastomotic leak. These satisfactory outcomes are comparable to those observed after proximal or extensive repair, stent grafting, or hybrid procedure performed at high-volume centers (2–5, 7, 9, 11, 13, 23).

In our opinion, the application of the Brain-Heart-first strategy in TAR with FET technique for patients with ADIAD may be able to end the dispute related to cerebral perfusion strategy (unilateral/bilateral and antegrade/retrograde cerebral perfusion) to some extent, and the arch replacement and cerebral protection are no longer the bottleneck. The surgical difficulties are transferred from the “arch” to the “root,” and a surgeon with skills to perform the Bentall procedure may be easily trained to perform this extensive repair. After the reconstruction/repair of the proximal aorta/aortic root, what remains to be done is the four routine graft-arterial anastomoses to reconstruct the arch and descending aorta, including the 2nd branch of the 4-branched graft with LCCA, the trunk of 4-branched graft with the stented graft, the 1st and 3rd branches of the 4-branched graft with LSA and IA, respectively. What's more, the procedure can be performed in a routine operation room with no need of expensive devices or complex operations involving interventional cardiologists and radiologists. For some complex cases or under particular circumstances, or for some surgeons who are not experienced enough, we suggest the Brain-Heart-first strategy be performed under moderate or even deep hypothermia to ensure patient safety. Therefore, we think it may be possible to transfer the results 1:1 to other centers, especially for high-volume centers.

In conclusion, the Brain-Heart-first strategy can be widely used with low technical and resource requirements and can provide a safe alternative for conventional TAR with FET technique in ADIAD patients with satisfactory operative results.

## Data Availability Statement

The raw data supporting the conclusions of this article will be made available by the authors, without undue reservation.

## Ethics Statement

The studies involving human participants were reviewed and approved by the Ethics Committee of the Second Xiangya Hospital. The patients/participants provided their written informed consent to participate in this study.

## Author Contributions

KS, LT, and HT: conception, study design, protocol, and writing. KS, LT, JX, DX, JL, SW, and YC: data collection and assessment. HT and XZ: project oversight and supervision. KS, LT, HT, and XZ: critical revisions for important intellectual content. All authors read and approved the final manuscript.

## Funding

This study was funded by the Key Research and Development Program of Hunan Province (No. 2019SK2022).

## Conflict of Interest

The authors declare that the research was conducted in the absence of any commercial or financial relationships that could be construed as a potential conflict of interest.

## Publisher's Note

All claims expressed in this article are solely those of the authors and do not necessarily represent those of their affiliated organizations, or those of the publisher, the editors and the reviewers. Any product that may be evaluated in this article, or claim that may be made by its manufacturer, is not guaranteed or endorsed by the publisher.

## Abbreviations

TAR: total arch replacement
FET: frozen elephant trunk
ADIAD: acute DeBakey type I aortic dissection
DHCA: Deep hypothermia circulatory arrest
CPB: cardiopulmonary bypass
HCA: hypothermic circulatory arrest
NIRS: near-infrared spectroscopy
LCCA: left common carotid artery
LSA: left subclavian artery
IA: innominate artery
ACC: aortic cross clamping
ACP: antegrade cerebral perfusion
LBA: lower body arrest
ICU: intensive care unit
RBC: red blood cells
FFP: fresh frozen plasma
CCR: creatinine clearance rate
NUML: multiples the upper normal limit
KDIGO: Kidney Disease Improving Global Outcomes
AKI: Acute Kidney Injury.
